# Johnson County Medical Society

**Published:** 1903-04

**Authors:** 


### Society Notes.

### Johnson County Medical Society.

The Johnson County Medical Society met March 13 at Cleburne, and disposed of the following program: Dr. Leake of Dallas read a paper on "Neurasthenia," published herein, which was discussed by all present. The society decided to have this paper published in Daniel's *Texas Medical Journal.* Paper by Dr. Graves of Georgetown on "Serum Therapy" was also discussed. The following officers were elected: Dr. W. P. Alexander, president; Dr. L. L. Harris, first vice president; Dr. R. H. Roark, second vice president; Dr. J. M. Huddleston, secretary. Drs. T. N. Self, E. B. Osborne, W. E. Menefee, a board of censors.

